# The Role and Modulation of Spinal Perineuronal Nets in the Healthy and Injured Spinal Cord

**DOI:** 10.3389/fncel.2022.893857

**Published:** 2022-05-20

**Authors:** Judith Sánchez-Ventura, Michael A. Lane, Esther Udina

**Affiliations:** ^1^Department of Cell Biology, Physiology and Immunology, Institute of Neuroscience, Universitat Autònoma de Barcelona, Barcelona, Spain; ^2^Centro de Investigación Biomédica en Red sobre Enfermedades Neurodegenerativas (CIBERNED), Bellaterra, Spain; ^3^Department of Neurobiology and Anatomy, College of Medicine, Drexel University, Philadelphia, PA, United States; ^4^The Marion Murray Spinal Cord Research Center, College of Medicine, Drexel University, Philadelphia, PA, United States

**Keywords:** perineuronal nets, CSPGs, spinal cord injury, plasticity, stability, ChABC

## Abstract

Rather than being a stable scaffold, perineuronal nets (PNNs) are a dynamic and specialized extracellular matrix involved in plasticity modulation. They have been extensively studied in the brain and associated with neuroprotection, ionic buffering, and neural maturation. However, their biological function in the spinal cord and the effects of disrupting spinal PNNs remain elusive. The goal of this review is to summarize the current knowledge of spinal PNNs and their potential in pathological conditions such as traumatic spinal cord injury (SCI). We also highlighted interventions that have been used to modulate the extracellular matrix after SCI, targeting the glial scar and spinal PNNs, in an effort to promote regeneration and stabilization of the spinal circuits, respectively. These concepts are discussed in the framework of developmental and neuroplastic changes in PNNs, drawing similarities between immature and denervated neurons after an SCI, which may provide a useful context for future SCI research.

## Introduction

The proper functioning of the nervous system strongly depends on a balance between plasticity and stability. On the one hand, plasticity is needed in both development and adulthood to constantly adapt to changing physiological demands and environmental stimuli. In contrast, stability is required to sustain appropriate network connectivity throughout life. In the mature nervous system, these two processes are dynamically controlled since their imbalance, in either direction, can lead to pathological outcomes (Takesian and Hensch, 2013). In the spinal cord, those spinal networks involved in the respiratory and locomotor control are a good example of this plasticity-stability trade-off. Both spinal circuitries produce stable and synchronized synapses to ensure the generation of an appropriate output: breathing or walking. Nevertheless, considering the environmental challenges that can perturb normal locomotion and respiration, a degree of flexibility is needed to permit correct reorganization of these networks, without leading to instability. This synaptic regulation is complex and not only requires the interplay of neurons and glial cells, but also the extracellular matrix (ECM), establishing the so-called tetrapartite synapse (Dityatev and Schachner, 2003; Dityatev and Rusakov, 2011). Particularly, there is a highly condensed ECM called the perineuronal net (PNN) that surrounds some neurons in the central nervous system (CNS) with a widely known role in synaptic stabilization. This reticular net dynamically controls plasticity and stability processes by rearranging their structure in an activity-dependent manner (Kalb and Hockfield, 1988; Dityatev et al., 2007). Although they were first described by Camillo Golgi in 1898, using a modified recipe of his silver impregnation technique (Celio et al., 1998), interest in PNNs was tempered until about a century later. Initially, his discovery was controversially discussed and finally dismissed by Ramón y Cajal who argued that PNNs were an artifact of his preparation. Thus, PNNs research was ceased until 1966, when histological techniques gradually improved and enabled researchers to visualize again PNNs using periodic-acid-Schiff (PAS) staining (Glegg and Pearce, 1956), lectins (Brauer et al., 1984), colloidal iron hydroxide (Seeger et al., 1994), and finally, specific antibodies to detect chondroitin sulfate proteoglycans (CSPGs) (Härtig et al., 1994). These histological advances helped to understand PNNs composition, but it was not until the twenty-first century that the role of PNNs in plasticity regulation was reported (Pizzorusso, 2002). Later, the interest in PNNs exponentially increased, and, in 20 years, PNNs have been linked to many other functions in the healthy and diseased brain.

Their presence in the spinal cord was firstly described in the cat in 1983 (Hockfield, 1983). Nevertheless, the function of spinal PNN was neglected compared with the encephalic ones. It is possible that the application of ChABC, an enzyme that degrades CSPGs, at the injured spinal cord to overcome the inhibitory environment created by the glial scar (McKeon et al., 1991) has blinded us to other roles of spinal PNNs under normal conditions due to ChABC impressive efficacy. Somehow, the presence of PNNs around denervated neurons far from the injury site becomes a barrier to axonal reinnervation and therefore, to functional recovery in SCI models (Massey et al., 2006; Alilain et al., 2011). However, recent research has revealed several differences between cortical and spinal PNNs regarding their composition (Vitellaro-Zuccarello et al., 2007; Irvine and Kwok, 2018), modulation (Smith et al., 2015), and the type of neurons surrounded by PNNs (Irvine and Kwok, 2018). These divergences have increased the interest in spinal PNNs and raised concern about the assumption that cortical and spinal PNNs play similar roles in both anatomical locations.

Overall, this review is focused on describing the role of PNNs in the CNS, highlighting the current knowledge of spinal PNNs and their relevance after an SCI.

## Perineuronal Nets

### Distribution

Perineuronal nets are specialized ECM that encapsulate the soma and the proximal dendrites of some neurons in the CNS. This mesh-like structure has been described in various mammals [e.g., rodents (Kalb and Hockfield, 1988; Galtrey et al., 2008), cats (Hockfield, 1983), dogs (Atoji et al., 1997), sheep (Härtig et al., 2017), primates (Mueller et al., 2016), and humans (Jäger et al., 2013)] and non-mammal species [e.g., fish (Takeda et al., 2018), birds (Balmer et al., 2009), frogs (Gaál et al., 2014), and chickens (Morawski et al., 2009)]. Among all of them, spinal PNNs have been characterized in fish, rodents, cats, primates, and humans.

Perineuronal nets are irregularly distributed throughout the brain (Brückner et al., 1996) and spinal cord (Vitellaro-Zuccarello et al., 2007). In the brain, they mainly ensheath fast-spiking, gamma-aminobutyric acid (GABA)ergic parvalbumin (PV) interneurons (extensively reviewed in Härtig et al., 1992; van't Spijker and Kwok, 2017). However, PNNs also surround pyramidal neurons in the hippocampus (Carstens et al., 2016), visual, somatosensory and motor cortex (Hausen et al., 1996; Alpár et al., 2006), PV-positive and PV-negative neurons in the striatum (Lee et al., 2012), and excitatory neurons in the amygdala and deep cerebellar nucleus (Carulli et al., 2006; Morikawa et al., 2017). PNNs' heterogeneity increases even more between species. For instance, while PNNs are mainly found in sensory brain areas in the rat, primates present a larger proportion of neurons with PNNs in motor areas (Mueller et al., 2016).

**Figure 1:**
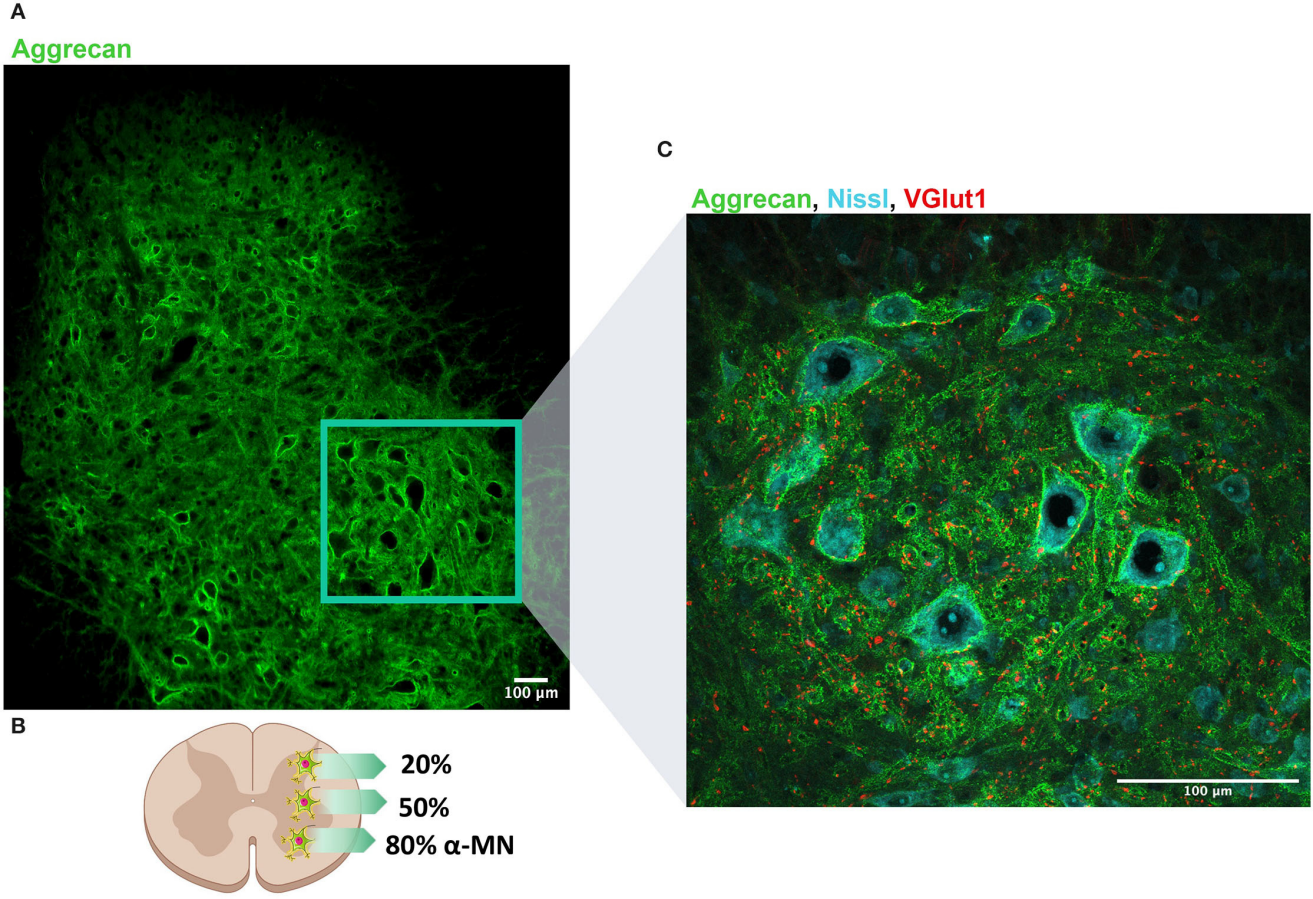
Perineuronal nets (PNNs) distribution along the dorso-ventral axis of the lumbar spinal cord. **(A)** PNNs are labeled by aggrecan (denoted in green) and observed along the whole lumbar spinal cord. **(B)** Schematic representation of the distribution of PNNs in the spinal cord: 20% of neurons in the dorsal horn present PNNs, whereas 50% of neurons located in the intermediate column are wrapped by PNNs. Considering α-MN, around 80% are surrounded by PNN. **(C)** Magnification of the region of the ventral horn where most of MNs are localized. Neurons are labeled in blue (Nissl) and proprioceptive afferents (VGlut1) that typically project to MN are labeled in red. Scale bar: 100 um.

Along the spinal cord, PNNs surround motoneurons (MNs) and spinal interneurons (Figure 1). Remarkably, there are differences in the proportion of PNN-enwrapped neurons throughout the spinal laminae (Figure 1B). In the dorsal horn, 20% of neurons present PNNs and specifically, none in the laminae I and II (Galtrey et al., 2008). The lack of PNNs in those laminae correlates with the grade of synaptic plasticity found in that region after injury, which is related to neuropathic pain development (Woolf et al., 1992). In contrast, in laminae VII and VIII, 50% of neurons have PNNs. This proportion has been associated with spinal interneurons including both PV- and calbindin-positive cells (Renshaw cells) (Vitellaro-Zuccarello et al., 2007). Finally, in contrast to the brain, PNNs located in the ventral horn surround large neural somas: spinal MNs (Vitellaro-Zuccarello et al., 2007; Galtrey et al., 2008). Notably, only α-MNs present PNNs in the spinal cord, unlike γ-MNs (Al'joboori et al., 2020). Possibly, the different PNNs expression around α- and γ-MNs could be explained by the electrophysiological properties of each MN type (Manuel and Zytnicki, 2019). Since PNNs formation during development is activity-dependent, the level of activity in each MN type seems to determine the presence or absence of this net. In fact, we have reported that vestigial PNNs modify the physiological properties of α-MNs (Sánchez-Ventura et al., 2021), suggesting a role in MN maturation. Considering the percentage of α-MNs enwrapped by PNNs, it was initially thought that only 30% of α-MNs had PNNs when they were stained with Wisteria floribunda lectin (WFA) (Galtrey et al., 2008). Nonetheless, selectively staining for the expression of aggrecan offers a different perspective on spinal PNNs, detecting around 80% of α-MNs with dense aggrecan-positive PNNs in rat (Irvine and Kwok, 2018) and 76% in primates (Mueller et al., 2016). These results certainly reinforce the physiological relevance of spinal PNNs.

### Composition and Structure

**Figure 2:**
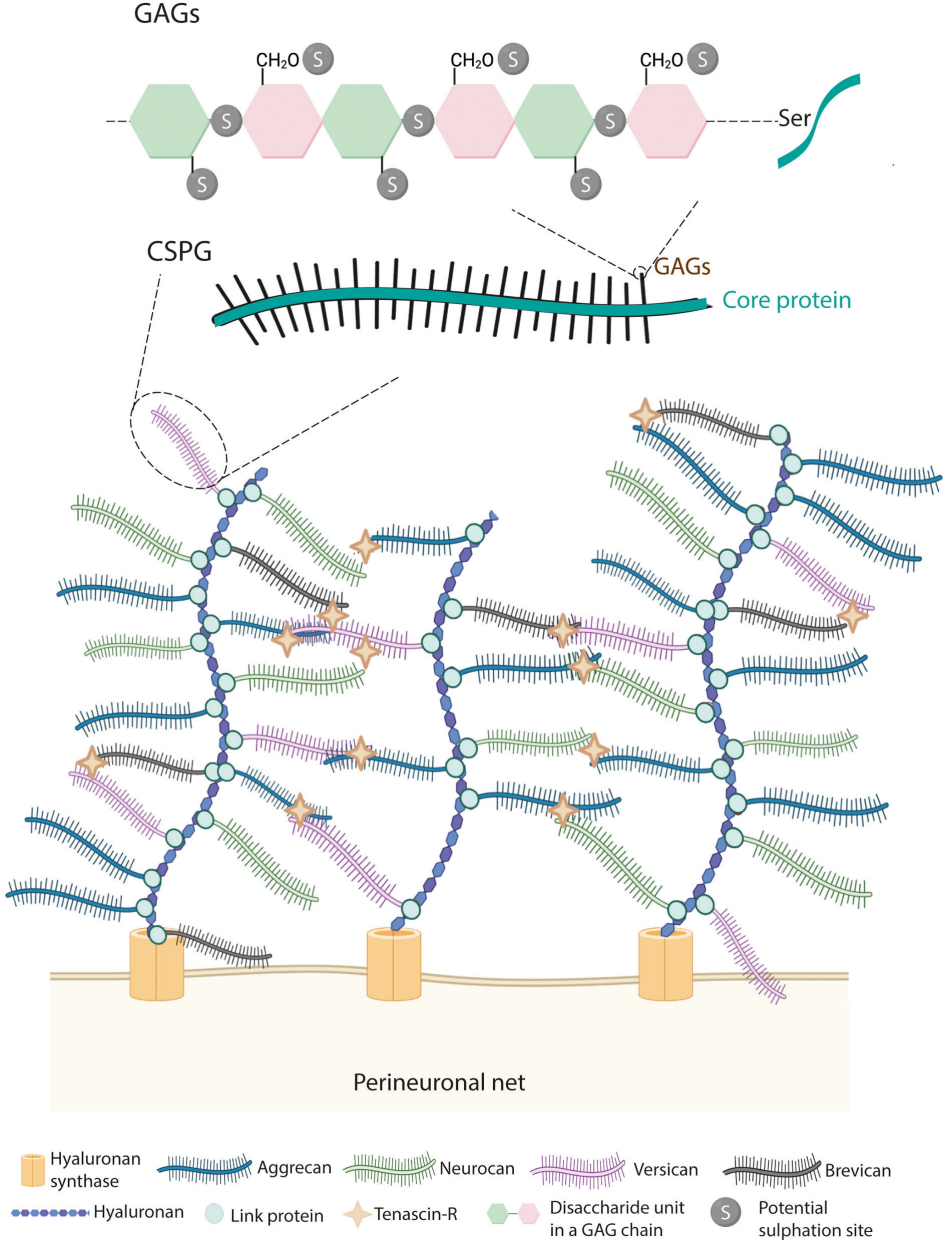
Structure of PNNs. PNNs are composed by hyaluronan, link proteins, chondroitin sulfate proteoglycans (CSPGs) and tenascin-R. Hyaluronan, secreted by the hyaluronan synthase (HAS), binds to members of the lectican family of CSPGs (i.e., aggrecan, brevican, versican, and neurocan) *via* link proteins. Then, tenascin-R further cross-links lecticans generating a lattice-like structure. CSPGs are formed by a core protein in which GAGs are covalently attached through serine residues. GAGs are composed of disaccharide units of chondroitin sulfate chains, which can be sulfated at the 2nd, 4th, and 6th positions.

Perineuronal nets are condensed ECM rich in CSPGs with holes at the site of synaptic contacts (Celio et al., 1998). PNNs are composed of hyaluronan (HA), CSPGs, link proteins, and tenascin-R (tn-R) (Kwok et al., 2011), which are highly organized in a ternary stable structure (Figure 2). PNNs' composition makes them different from the diffuse ECM, widely present throughout the CNS. Although both types of ECM contain HA, tn-R, and some CSPGs, the diffuse matrix lacks link proteins.

Among all PNNs components, link proteins are crucial for their formation. *In vitro*, the lack of the Crtl1 gene, which encodes the link protein 1, prevents PNNs formation around PNN-bearing cells (Kwok et al., 2010). *In vivo*, it generates attenuated PNNs in the visual cortex (Carulli et al., 2010) and aberrant PNNs in the spinal cord, with an altered proportion of its components (Sánchez-Ventura et al., 2021). Another important element in PNNs structure is aggrecan (Rowlands et al., 2018). While mice deficient for the lecticans neurocan (Zhou et al., 2001) or brevican (Brakebusch et al., 2002) present organized PNNs, aggrecan-deficient mice show altered PNNs with no WFA staining (Giamanco et al., 2010). The importance of tn-R in PNNs formation is observed in the tn-R knockout (KO) mouse whose PNNs structure is affected in both development and adulthood (Weber et al., 1999; Brückner et al., 2000; Haunso et al., 2000).

Most PNNs components, including aggrecan, hyaluronan synthase (HAS), and link proteins (Matthews et al., 2002), are produced by neurons, although astrocytes and oligodendrocytes also contribute to PNNs formation through synthesizing neurocan (Jones et al., 2003) and tn-R (Galtrey et al., 2008), respectively. PNNs are anchored to neurons by the enzyme HAS, which produces an HA polymer chain on the neuronal surface, constituting the backbone of PNNs. This conclusion was reached in view that HA receptors such as CD-44 are expressed in glial cells but not in neurons (Aruffo et al., 1990), dismissing the idea that HA receptors are the link between PNNs and neurons and accepting that HAS are the ones that attach PNNs to the neural membrane. In the spinal cord, HAS 3 is expressed in PNN-enwrapped neurons located in both the dorsal and ventral horn in adulthood. In contrast, HAS 1 is only expressed in the developing spinal cord (Galtrey et al., 2008).

Hyaluronan provides a scaffold for the binding of CSPGs. CSPGs are the major components of PNNs representing only 2% of all the CSPGs of the nervous system (Fawcett, 2015). Among CSPGs, aggrecan is the major constituent of spinal PNNs, and three other CSPGs (i.e., brevican, neurocan, and versican) are present in PNNs in variable degrees. All types of CSPGs are composed of two parts, namely, a core protein and a variable number of glycosaminoglycan (GAG) chains. The core proteins are a tridomain structure formed by a N- and C-terminal globular domain, necessary for the binding of HA and tn-R, respectively, and a central region (Yamaguchi, 2000). In the N-terminal, the interaction between HA and CSPGs is maintained by link proteins (HAPLN family genes). Although HAPLN1, 2, and 4 are found in the nervous system, in the spinal cord, only HAPLN1/Crtl1 and HAPLN4/Bral2 are described in PNN-bearing neurons (Galtrey et al., 2008). Tn-R binds to the C-terminal of the CSPGs and, thanks to its trimeric structure, facilitates the cross-link between HA and CSPGs (Lundell et al., 2004). In the central region, GAGs are covalently attached to the core protein through serine residues.

Although PNNs disposition is quite preserved, slight changes in CSPGs conformation contribute to PNNs heterogeneity. Their heterogeneity arises from the type of core protein found (Dauth et al., 2016) and/or the number and sulfation pattern of GAG chains (Miyata and Kitagawa, 2017). Antibodies against different CSPGs core protein showed a different distribution compared with the brain and spinal cord sections (Galtrey et al., 2008). The heterogeneity of the aggrecan core protein can increase even more since different glycosylation variants of this lectican also display differential distribution in different neuronal types in the CNS (Matthews et al., 2002). The GAGs can be sulfated at different positions including the 4th (CS-A), 6th (CS-C), 2nd−6th (CS-D), and 4th−6th (CS-E). Changes in this sulfation pattern can modify CSPGs charge and thus, provide binding properties to PNNs, a characteristic that also differs from the diffuse ECM (Carulli et al., 2006). Interestingly, CS-A and CS-C have different characteristics, while CS-A is inhibitory to axon growth and suppresses plasticity, CS-C is permissive to axon growth and increases plasticity (Wang et al., 2009; Miyata et al., 2012). Furthermore, this sulfation pattern may vary depending on the CNS region since WFA, which detects the non-sulfated GalNAc residues (Nadanaka et al., 2020), shows a low percentage of detection around spinal PNNs (Irvine and Kwok, 2018) compared with that in cortical ones.

### Function

Although PNNs are widely known for their role in plasticity inhibition and synaptic stabilization, other functions have been attributed to cortical PNNs such as ionic buffering, neuroprotection, and neural maturation (extensively reviewed in Kwok et al., 2011; van't Spijker and Kwok, 2017; Lorenzo Bozzelli et al., 2018; Fawcett et al., 2019). PNNs functions are tightly related to the structural properties of their CSPGs. Hence, manipulation of PNN's CSPGs has a direct impact on the function of PNNs and the neuron surrounded (Gama et al., 2006). The highly negative charge of GAGs is one of the most important factors determining PNNs functions (Brückner et al., 1993), and this can be considered in several different ways:

- PNN's negative charges provide a suitable microenvironment around fast-firing neurons due to their buffering capacity of local ions. PNNs can control the diffusion of ions serving as a fast cation exchanger to provide rapid neuronal responses (Härtig et al., 1999). Besides, in the brain (Härtig et al., 1999; Carulli et al., 2006) and the dorsal and intermediate zone of the spinal cord (Deuchars et al., 2001), there is a good correlation between the expression of the potassium channel Kv3.1b, a marker of fast-firing neuron (Rudy and McBain, 2001) and PNNs. Interestingly, this is not the case for spinal MNs, and thus, PNNs function in these neurons needs to be further investigated.
- The negative charges of GAGs can buffer cations produced after oxidative stress or toxic metal ions, conferring neuroprotective capabilities to PNNs (Morawski et al., 2010; Suttkus et al., 2014).
- The negative milieu, provided by the charge, around neurons determines the membrane capacitance, which affects neural excitability (Tewari et al., 2018). Indeed, Glykys et al. (2014) found a negative correlation between PNNs intensity and internal Cl^−^ concentration, suggesting that PNNs are involved in setting the local Cl^−^ levels. Indeed, PNNs degradation resulted in increased neural excitability (Hayani et al., 2018).
- Negative charges can, directly and indirectly, inhibit neural regeneration. Although the mechanisms used by CSPGs to inhibit neural regeneration are not completely understood, the GAG chains generate a steric hinderance for regrowth. This is observed in the fibro-glial scar formed after an SCI (McKeon et al., 1999) and overcome after ChABC application (Bradbury et al., 2002). Indirectly, their inhibitory properties can also be triggered after interacting with specific receptors (Shen et al., 2009; Lang et al., 2015). Moreover, GAGs chains disulfated at the 4th−6th position facilitate the binding of the repulsive guidance molecule semaphorin 3A (Sema3A), which inhibits neural outgrowth and regeneration (Dick et al., 2013, Vo et al., 2013).

Importantly, the sulfation pattern can contribute to other neural functions such as maturation. After the binding to the PNNs through CS-E chains, the Otx2 protein can translocate into neurons and facilitate PV positive-neurons maturation (Sugiyama et al., 2008; Beurdeley et al., 2012). PNNs components also interact with ion channels and receptors and thus, regulate synaptic activity and membrane current. Tn-R binds to GABA receptors (GABAr) through the HNK-1 motif (Saghatelyan et al., 2000). This PNN component also interacts with subunits of voltage-gated Na^+^ channels (Xiao et al., 1999), while brevican interacts with K^+^ channels and α-amino-3-hydroxy-5-methyl-4-isoxazolepropionic acid receptor (AMPAr) (Favuzzi et al., 2017). Consequently, their removal can alter the excitatory/inhibitory balance. Furthermore, the lateral mobility of some receptors is also restricted by PNNs. ECM digestion with ChABC increases the mobility of AMPAr (Frischknecht et al., 2009).

Overall, while PNNs have been classically defined as a barrier for plasticity, they also regulate numerous different neuronal functions. These functions that appear to be crucial to neural development may play adaptive or maladaptive roles after SCI, and effort is needed to improve our understanding of these functions in the spinal cord.

## Development: PNN Formation

During postnatal development, there is a window of plasticity, called the critical period, during which neural circuits are sensitive to environmental stimuli (Hubel and Wiesel, 1970; Berardi et al., 2000). This sensory experience increases neuronal activity resulting in a boost of plasticity that favors synaptic wiring and fine-tunes spinal neuron properties (Kalb and HockField, 1994; Cameron and Núez-Abades, 2000). During this period, excitatory circuits predominate over inhibitory ones, as the high Cl^−^ levels within immature GABAergic neurons result in depolarizing responses. However, this sensory experience progressively produces some intracellular and extracellular changes around immature neurons. On the one hand, it gradually lowers Cl^−^ inside GABAergic neurons, switching from depolarizing to hyperpolarizing actions (Ben-Ari et al., 2012). The developmental upregulation of the KCC2 K^+^/Cl^−^ cotransporter also contributes to this shift (Rivera et al., 1999). In contrast, it progressively increases the expression of some PNNs components until reaching a peak in which the link protein 1 is upregulated and condensed PNNs appear (Carulli et al., 2010). At that time point, there is also an increase in the sulfation CS-A/CS-C ratio of PNNs' CSPGs (Carulli et al., 2006; Miyata et al., 2012). This increase is comparable among the three spinal regions, although it appears earlier in the cervical segment than in the thoracic and lumbar one (Takiguchi et al., 2021). In the spinal cord of rodents, PNNs formation occurs in the second postnatal week (Kalb and Hockfield, 1988; Galtrey et al., 2008), marking the end of the critical period for plasticity. In fact, digestion of PNN by the enzyme ChABC reopens this window of plasticity (Pizzorusso, 2002; Pizzorusso et al., 2006). Indeed, the GABAergic shift occurs at the end of the critical period too, coinciding with PNNs deposition around neurons (Berardi et al., 2000; Frischknecht et al., 2009; Takesian and Hensch, 2013). A negative correlation between PNNs intensity and intracellular Cl^−^ levels was reported and further confirmed after digesting PNNs and measuring increased intracellular Cl^−^ levels (Glykys et al., 2014).

Once PNNs are fully formed, they mold the new connections generated into a meaningful manner to prepare the circuitry for adulthood: while active neurons would be wrapped by PNNs to strengthen their connections, their absence around unused synapses would lead to their pruning (reviewed in Murakami et al., 1992). In the spinal cord, around 50% of synapses are lost during development (Ronnevi et al., 1974). Interestingly, transgenic mice lacking the Crtl1 gene have aberrant PNNs and an increased number of excitatory synapses around spinal MNs (Sánchez-Ventura et al., 2021). Likewise, transgenic mice lacking tn-R, neurocan, and brevican also exhibited an increase in excitatory synapses around cortical neurons (Silver and Miller, 2004). Therefore, an adequate synaptic balance in mature neurons depends on the presence of proper PNNs.

**Figure 3:**
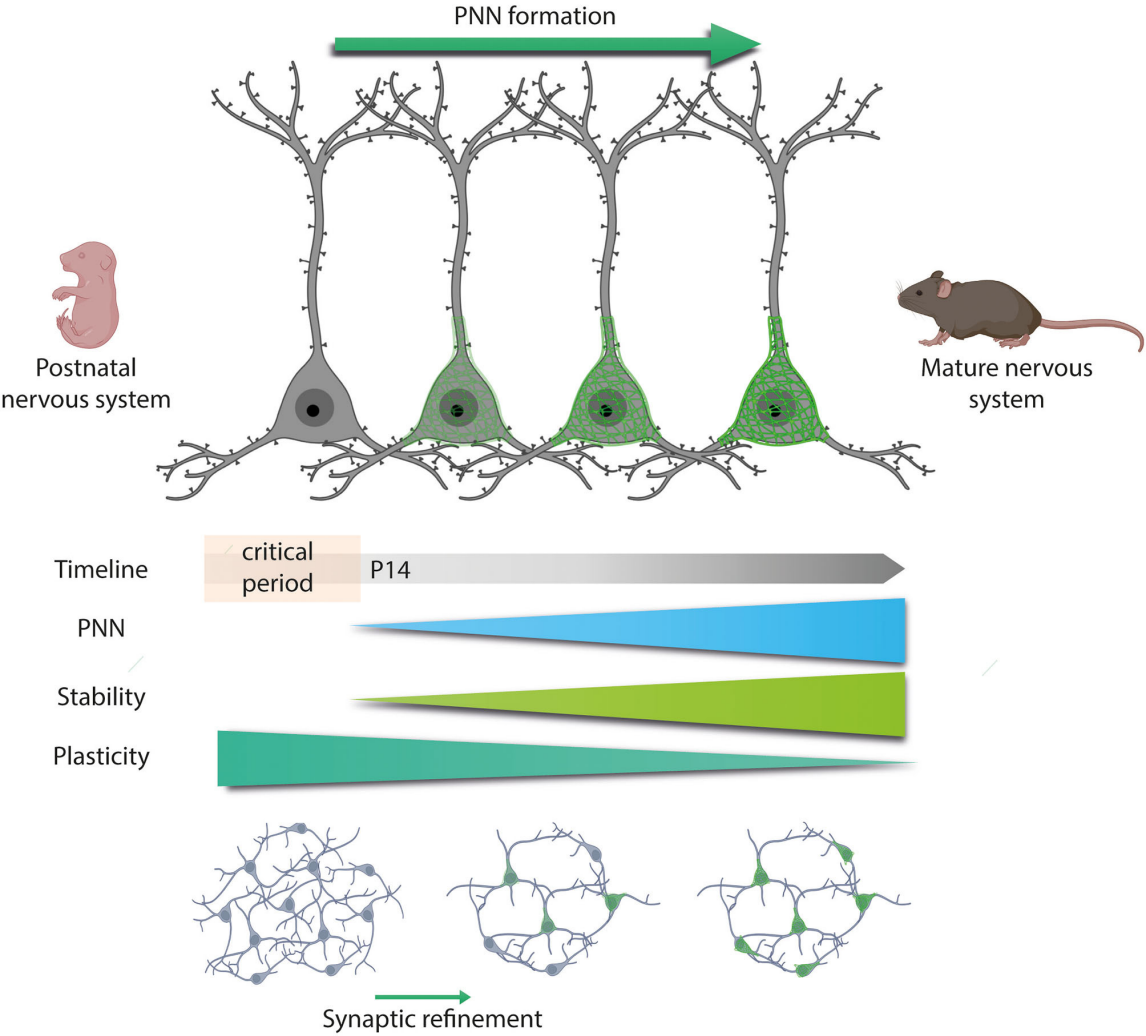
Developmental PNNs formation. PNNs first appear during development, specifically at the end of the critical period, P14 in the spinal cord. The critical period is a plastic phase in which neurons increased their activity and generate new synaptic contacts. Once PNNs appear, they would wrap active neurons and stabilize these new connections formed. However, the absence of PNNs around unused synapses would lead to their pruning. Thus, as long as PNNs are formed, the plasticity of the central nervous system (CNS) decreases while stability increases.

Thus, the formation of PNNs is activity-dependent and coexists with the end of the critical period and the maturation of the CNS in which synaptogenesis, synaptic refinement, and neural maturation occur (Figure 3) (Pizzorusso, 2002; Carulli et al., 2006; Galtrey et al., 2008). Alterations in this activity can produce changes in the morphology, connectivity, and electrophysiological properties of MNs. In the spinal cord, all the excitatory drive that MNs received during development is provided by proprioceptive and supraspinal inputs (Kalb and HockField, 1994). Hence, sciatic nerve injury or thoracic hemisection before PNNs deposition suppresses MN activity and disrupts aggrecan expression (Kalb and Hockfield, 1988). Consequently, this reduced MN activity decreases MN pruning (Pittman and Oppenheim, 1979) and size (Brandenburg et al., 2018). Reduced MN size during development changes MN excitability and consequently affects motor neuron recruitment, since small MN cell bodies are more excitable and are recruited before large MNs (Henneman et al., 1965), directly impacting neural circuits (Kalb and HockField, 1994).

Overall, PNNs are instrumental in the transition from a permissible to a restricted milieu in the adult. The GABA potential shift and PNNs formation contribute to the well-known inhibitory environment found in the mature nervous system.

## Mature Nervous System: PNN Function and Modulation

Once the critical period ends and PNNs have fully emerged, plasticity is not permanently lost but rather regulated more rigorously under the dynamic control of PNNs. This dynamism is explained by the activity-dependent modulation of PNNs still present in the adult: changes in the activity of mature neurons would structurally rearrange PNNs, and consequently, plasticity would be restricted or facilitated (Smith et al., 2015; Favuzzi et al., 2017). This modulation is mediated by endogenous and exogenous mechanisms. Exogenous mechanisms are mainly used in experimental studies and consist in the application of degrading enzymes like ChABC and hyaluronidase (Pizzorusso, 2002; Massey et al., 2006; Pyka et al., 2011; Starkey et al., 2012). Endogenous mechanisms are the main physiological modulators of PNNs, adjusting the synthesis and degradation of the different PNNs components. This constitutive remodeling is controlled by several protease families such as metalloproteases (MMPs) and A Disintegrin and Metalloproteinase with Thrombospondin motifs (ADAMTS) (Cawston and Young, 2010). Furthermore, the sulfation of CSPGs is another endogenous way through which activity can alter PNNs condensation and conformation (Miyata et al., 2012).

The regular turnover of PNNs persists throughout life, and it is crucial for many physiological processes. Interestingly, activity differently modulates PNNs depending on their anatomical location (Smith et al., 2015). In the cerebellum, an enriched environment (EE) reduced the synthesis of PNNs components and enhanced their degradation by increasing the activity of MMP2 and MM9 (Foscarin et al., 2011). In contrast, in the spinal cord, Wang et al. (2011) were the first to demonstrate that activity, in terms of rehabilitation, increased spinal PNNs in an SCI model. This specific finding, the impact of physical activity on spinal PNNs, did not receive much attention, despite clearly demonstrating that activity differentially modulates spinal and encephalic PNNs. Afterward, two studies corroborated that physical exercise increased PNNs around intact spinal MNs (Arbat-Plana et al., 2015; Smith et al., 2015). Thus, the same physical activity that decreases PNNs' thickness around neurons located in the brain increases PNNs' thickness in the spinal cord (Smith et al., 2015). This differential activity-dependent modulation could have a biological significance, as most spinal cord functions rely on the stability of spinal circuits, whereas plasticity is essential for most brain functions. The relevance of spinal reflexes' stability in spinal cord function has been demonstrated in transgenic mice with aberrant PNNs that display motor impairment (Sánchez-Ventura et al., 2021).

Finally, the malleability of PNNs maintains the nervous system in an equilibrium whose imbalance in any direction can have broad implications in terms of neurological diseases. Excessive proteolytic processing of PNNs can lead to excessive plasticity as well as increased vulnerability to neurotoxic stimuli, which can trigger neurological disorders mainly studied in the brain. Indeed, cortical PNNs alterations have been linked with seizures (Tewari et al., 2018), CNS infection (Belichenko et al., 1997), traumatic brain injury (Hsieh et al., 2017), and stroke (Hobohm et al., 2005), among others. Nonetheless, very few studies have evaluated whether changes in PNNs can lead to pathological conditions in the spinal cord.

## Injured Nervous System: Spinal Cord Injury

Similar to the brain, insults to the spinal cord such as a traumatic SCI result in alterations in the ECM that consequently break the plasticity-stability balance. The most evident changes in ECM following SCI are related to the formation of the fibro-glial scar at the injury site, mainly produced by astrocytes and fibroblasts. However, less is known about the changes that spinal PNNs suffer due to the injury and the impact of these alterations on spinal circuits.

### Fibro-Glial Scar in the Injury Site

After an SCI, there is an upregulation of CSPGs, at the injury site, forming the so-called fibro-glial scar. Specifically, there is a significant increase in lecticans such as neurocan, versican, phosphacan, NG2, and brevican but not aggrecan (Lemons et al., 2001; Jones et al., 2003; Buss et al., 2009). These CSPGs persist in the glial scar (Silver and Miller, 2004) and are remodeled by MMP, which expression significantly increases after injury (de Castro Jr et al., 2000; Zhang et al., 2011). Apart from CSPGs upregulation, selective changes in the sulfation pattern of GAGs have been reported. Following SCI, a large increase in the 4-sulfated (CS-A) GAG chains in the injury site have been observed, with no changes in the 6-sulfated (CS-C) GAG ones (Wang et al., 2009; Hussein et al., 2020).

The glial scar cannot be simply defined as beneficial or detrimental to CNS repair since it walls the injured area but also forms a barrier for axonal regeneration (Fawcett and Asher, 1999; Sofroniew, 2010). A lot of effort has been made to find an effective strategy to overcome the inhibitory influence of GAGs and thus, promote regeneration. These strategies are extensively exposed in the last section.

### Spinal PNN After SCI

**Figure 4:**
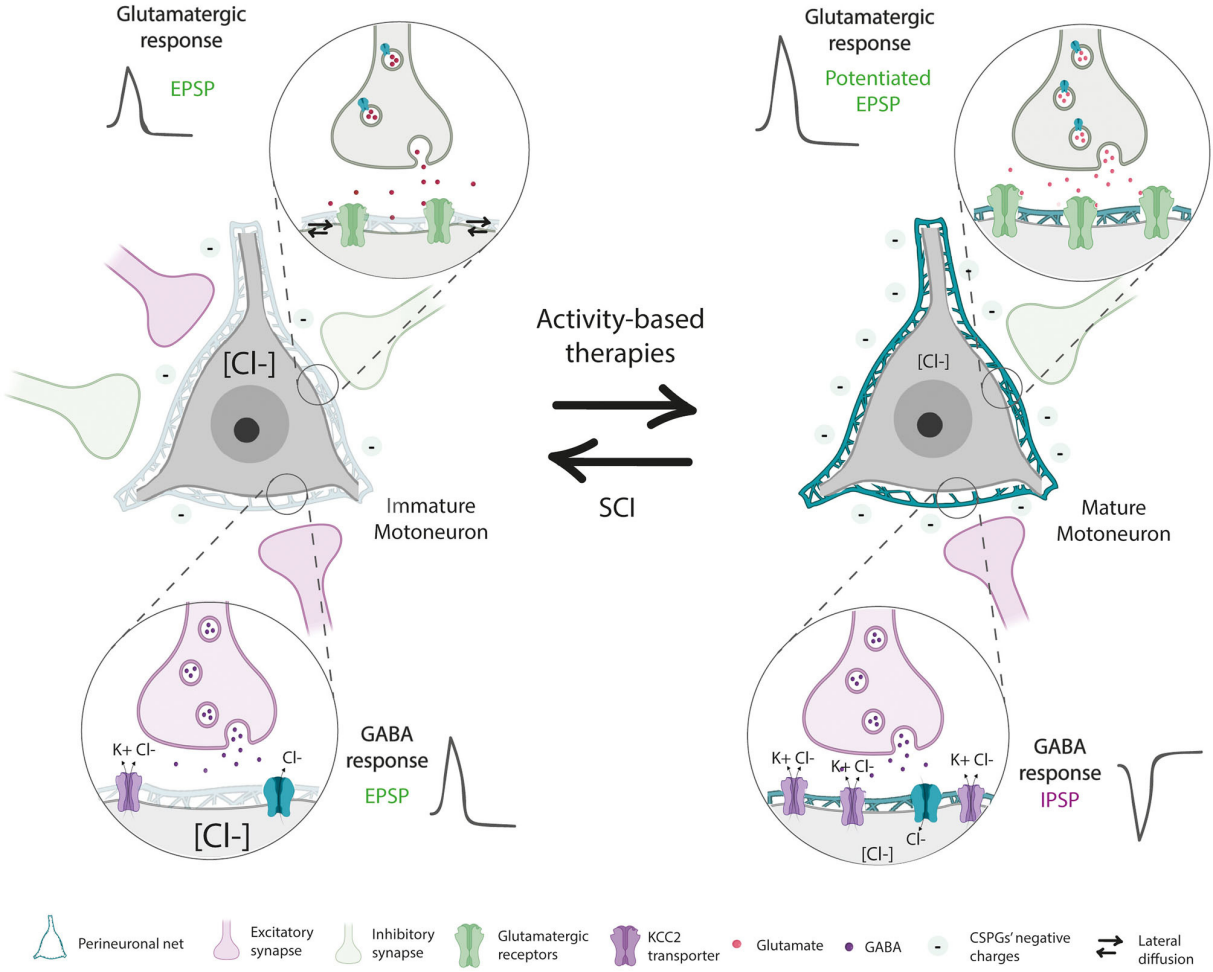
Developmentally immature neurons and denervated neurons post-spinal cord injury (SCI) share similarities in PNN integrity and neuronal properties. Mature neurons present stable PNN, which generates a high density of negative charges around neurons that maintain ionic homeostasis necessary for proper synapse functioning. Besides, the physical barrier provided by PNNs maintains receptors in the cell surface and stabilizes synapses. However, after SCI, denervated neurons reduced PNNs thickness, shifting the GABAergic response of inhibitory neurons due to a reduction of the KCC2 co-transporter and a decrease of their negative chargers around the neuron. Besides, PNNs decline facilitates the mobility of receptors, generating inefficient excitatory synapses. These characteristics resemble those seen in developmentally immature neurons. The application of activity-dependent therapies prevents PNNs reduction and restores mature neuron properties.

The fate of PNNs located below the level of the injury is controversial since different studies have reported a reduction (Lemons et al., 2001), no changes (Al'joboori et al., 2020) or an increase (Alilain et al., 2011) in PNNs thickness. Alilain et al. (2011) showed an upregulation of CSPGs, labeled by WFA, associated with PNNs around phrenic MNs after a cervical SCI. However, they did not specifically quantify PNNs around the neuronal soma, which is needed to differentiate PNNs from the loose ECM found in the grey matter. In addition, since an upregulation of CSPGs in the brainstem was also observed after a dorsal column section (Massey et al., 2006), it was assumed that SCIs increase PNNs from any type of neuron denervated by the injury, reducing plasticity and limiting axonal regeneration. Later studies tried to address the fate of spinal PNNs far from the injury site. Reduced staining of aggrecan around lumbar MN was reported 35 days after a thoracic SCI, recovering normal levels at later stages (Sánchez-Ventura et al., 2020). Similar findings were described after hemisection in goldfish (Takeda et al., 2018). A study evaluating aggrecan synthesis and degradation found a significant decline in aggrecan levels after SCI too (Lemons et al., 2001). In contrast, a recent study described no change in the amount of WFA+ CSPGs in the lumbar ventral horn after a thoracic SCI at early time points. However, at 67 days post injury, a significant increase was found (Al'joboori et al., 2020). Taken together, these findings point to some controversy regarding the fate of spinal PNNs after SCI not only because of the marker used to assess CSPGs' changes (WFA vs. aggrecan) but also to the type of neuron evaluated. PNNs surrounding phrenic MNs can behave differently than lumbar ones, given their differing functions (breathing vs. locomotion) and innervation [low vs. high proprioceptive Ia afferents (Alvarez et al., 2004; Nair et al., 2017)]. Besides, neurons in the brainstem are not located in the spinal cord, and hence, their PNNs can present the same activity-dependent modulation than encephalic PNNs (Sánchez-Ventura et al., 2020). Finally, the chronicity and type of injury may also impact on PNNs modulation. Long-term changes can be masked by the spontaneous recovery observed in some SCI models. Indeed, the progressive locomotor recovery in injured animals can modulate PNNs, since physical activity increases PNNs around spinal MNs (Wang et al., 2011; Arbat-Plana et al., 2015; Sánchez-Ventura et al., 2020). This increase is linked to the type and intensity of the activity (Arbat-Plana et al., 2015, 2017; Sánchez-Ventura et al., 2020), as well as the ability of the animal to be active besides the physical protocol (Arbat-Plana et al., 2015, 2017; Al'joboori et al., 2020). Thus, the denervated state of neurons below the injury produced by the disruption of descending inputs and proprioceptive inactivity due to muscle paresis might reduce PNNs, reverting neurons to their developmental state before PNNs first appear. In fact, PNNs reduction after injury is accompanied by changes that resembled those seen during the critical period, including increased synapse formation and altered neuronal excitability (Arbat-Plana et al., 2015; Sánchez-Ventura et al., 2020). Hence, developmentally immature neurons and denervated neurons post-SCI share similarities in PNNs integrity and neuronal properties (Figure 4). Lumbar PNNs disintegration after SCI was accompanied by reduced expression of the KCC2 co-transporter (Sánchez-Ventura et al., 2020), probably due to the role of PNNs sustaining receptors in the cell membrane (Frischknecht et al., 2009), modifying Cl^−^ intracellular levels. In parallel, the loss of negative charges provided by the CSPGs of spinal PNNs can shift the GABAergic response of spinal neurons and thus, increase spinal excitability (Sánchez-Ventura et al., 2020). Therefore, the increased collateral sprouting and neuron excitability observed after SCI (review in Oudega and Perez, 2012) are also reported in transgenic mice with aberrant PNNs. Animals lacking the link protein 1 presented an altered ratio of excitatory and inhibitory synapses leading to hyperexcitability of spinal circuits. After SCI, these animals presented increased sprouting of the corticospinal tract (CST) (Sánchez-Ventura et al., 2021), corroborating that spinal PNNs play an important role in stabilizing synapses and limiting plasticity in the mature nervous system. Overall, while the absence of PNNs before the end of the critical period is necessary for developmental synaptogenesis, PNNs reduction after SCI can lead to uncontrolled sprouting and hyperexcitability in the spinal cord that leads to maladaptive symptoms such as neuropathic pain and spasticity (Costigan and Woolf, 2000; Boulenguez et al., 2010; Pitcher and Cervero, 2010; Côté et al., 2014; Mòdol et al., 2014). Interestingly, the blockage of MMP-9 and MMP-2 in the spinal cord inhibits the early and late phases of neuropathic pain, respectively (Kawasaki et al., 2008).

Finally, altered spinal PNNs are also observed in other pathologies such as amyotrophic lateral sclerosis (ALS) and spinal muscular atrophy (SMA). Indeed, disorganized and vestigial PNNs are found in the ventral horn of terminal SOD1 mice (Forostyak et al., 2014), an experimental model of ALS. *In vitro* and *in vivo* studies revealed that patients with SMA present a downregulation of the Ctrl1 gene, altering their PNNs (Dayangac-Erden et al., 2018).

### Therapeutical Manipulations of the ECM After SCI

The application of the enzyme ChABC in the fibro-glial scar is one of the most successful strategies to overcome the inhibitory influence of GAGs and consequently, promote regeneration (McKeon et al., 1991). The effectiveness of ChABC in degrading CSPGs have been tested *in vitro* and *in vivo* (Lee et al., 2010). *In vivo*, most of its efficacy has been evaluated in rodents, though it has also been studied in larger mammals such as cats and non-human primates (Tester and Howland, 2008; Rosenzweig et al., 2019). In most cases, ChABC has demonstrated huge potential in promoting regeneration of dopaminergic (Moon et al., 2001) and sensory axons (Shields et al., 2008), as well as, inducing sprouting of both intact and injured serotoninergic (Tom et al., 2009), corticospinal (Barritt et al., 2006; García-Alías et al., 2009), and sensory fibers (Massey et al., 2006). However, controversial results do appear in the literature too. When CST regeneration was compared in hemisected and contusion models, ChABC injections only enhanced regeneration of the CST in the hemisection model (Iseda et al., 2008). Accordingly, ChABC's effect may depend on the severity and location of the lesion, in addition to the number of spared axons and the time of the ChABC injection. Indeed, Warren et al. (2018) described that ChABC was more effective when applied chronically than acutely after a cervical hemisection.

Although axonal growth (regeneration or sprouting) is typically necessary for recovery, it is not always sufficient. Despite some studies have demonstrated improved motor, sensory and bladder function after ChABC application (Bradbury et al., 2002; Caggiano et al., 2005; Massey et al., 2006; Cafferty et al., 2008), many others found limited results. The administration of a Sema3A inhibitor enhanced axon regeneration but not recovery due to a lack of functional connections (Zhang et al., 2014). Similarly, some works have reported limited recovery after ChABC application since the plasticity promoted by this enzyme needs an appropriate interaction with their target to make functional networks (García-Alías et al., 2009; Tom et al., 2009; Harris et al., 2010; Alilain et al., 2011; Wang et al., 2011).

Indeed, the combination of ChABC or Sema3A inhibitor with specific rehabilitation further enhanced functional recovery compared with applying them alone. In this regard, it has been proposed that the plasticity achieved by ChABC or Sema3A inhibitor only establishes meaningful connections when is combined with rehabilitative training, which activates spinal circuits, increases spinal PNNs around active neurons (Arbat-Plana et al., 2015; Smith et al., 2015; Sánchez-Ventura et al., 2020), and stabilizes these connections (García-Alías et al., 2009; Wang et al., 2011; Zhang et al., 2014). The ability of rehabilitation to rewire and stabilize synapses in a functionally meaningful manner after SCI has been already reviewed (reviewed in Torres-Espín et al., 2018). Nevertheless, many questions remain unanswered regarding the mechanisms involved. Since this synaptic stabilization is comparable to that found at the end of the critical period, in which PNNs contribute, it is plausible that these nets participate in the recovery promoted by rehabilitation after SCI. As stated before, activity increases spinal PNNs' thickness, in contrast to the brain (Smith et al., 2015). Physical activity and EE following SCI resulted in increasing spinal PNNs around lumbar MN whereas reducing PNNs in the brainstem sensory nuclei (Sánchez-Ventura et al., 2020). At the spinal level, physical activity prevented PNNs decline caused by the injury (Wang et al., 2011; Arbat-Plana et al., 2015; Smith et al., 2015; Sánchez-Ventura et al., 2020), which probably contributes to the synaptic stabilization of the newly formed connections and hence promotes functional recovery (García-Alías et al., 2009; Wang et al., 2011; Zhang et al., 2014; Sánchez-Ventura et al., 2020).

The importance of PNNs integrity after SCI is also observed in the CST. Digestion of cortical PNNs located in the boundary between motor and sensorimotor cortex by ChABC perturbed anatomical and functional CST reorganization after injury, resulting in an aggravation of motor deficits (Orlando and Raineteau, 2015).

Overall, combinatorial therapies appear to be the most effective approach to enhance functional recovery after an SCI. This combination can include therapies to widen the window of plasticity and modify the glial scar, such as the enzyme ChABC, and rehabilitation to shape the newly formed connections. However, the application of this enzyme can indirectly digest spinal PNNs and entail deleterious effects on the physiology of spinal neurons. Therefore, the ChABC approach presents limitations in the context of human therapy not only due to its non-specific nature but also due to the multiple injections or large volumes needed. Hence, more precise and selective manipulations than ChABC have been proposed to enhance axonal regeneration. Transgenic mice expressing ChABC under the GFAP promoter, thus limiting ChABC expression on astrocytes, showed enhanced corticospinal regeneration at the injury site (Cafferty et al., 2007). However, given that astrocytes can also contribute to PNNs' turnover, this type of approach can also indirectly act on spinal PNNs. Similarly, targeting the mRNA of critical enzymes in CSPGs' glycosylation and elongation has been tested *in vitro* (Grimpe and Silver, 2004; Laabs et al., 2007) and *in vivo* (Grimpe and Silver, 2004) with encouraging results in regeneration around the lesion site. However, the selectivity of these molecules in the glial scar is unclear. To solve this non-specificity, antibodies against specific structures are found in the literature. On the one hand, antibodies that neutralize the CSPG NG2 have demonstrated effectiveness in increasing regeneration of sensory axons after injecting into the dorsally transected spinal cord (Tan et al., 2006). Considering that no work has previously described the presence of the lectican NG2 in spinal PNNs, the application of this neutralizing antibody could offer an alternative way to specifically target the glial scar (Galtrey et al., 2008; Irvine and Kwok, 2018). In contrast, the application of selective antibodies against 4-sulfated GAG chains improved neurite growth (Yang et al., 2017), suggesting that modulating the sulfation pattern of proteoglycans can be an alternative to ChABC. In fact, while 4-sulfated GAG is distinguished by its inhibitory properties, the 6-sulfated GAG can also be beneficial for regeneration (Gilbert et al., 2005).

The therapeutic modulation of the PNNs must strike an accurate balance. Widely, PNNs digestion can generate excessive plasticity and increase neural vulnerability to neurotoxic stimuli, whereas excessive PNNs deposition can generate increased levels of synaptic stability. Apart from the modulation provided by activity-dependent therapies such as rehabilitation or exposure to an enriched environment, PNNs can be also modulated pharmacologically. In this sense, targeting specific PNNs components, such as Sema3A protein or the sulfation patterns of GAGs, are promising approaches.

## Closing Remarks

Growing evidence sheds light on the potential of PNNs in controlling the proper function of the CNS, especially in the brain. However, more spinal cord research is needed to improve our understanding of spinal PNNs dynamics and function. It will be important to clearly define functional changes in the PNNs, the loose ECM, and the glial interface that accumulate in injured tissues, to completely understand the consequences of non-specific CSPG digestion. Digestion with ChABC alone does not reveal the full extent to which spinal PNNs can adaptively or maladaptively regulate changes after SCI, since only 2% of all CSPGs are found in PNNs (Fawcett, 2015). Future work harnessing the strengths of transgenic animals with aberrant PNNs, specifically those lacking the Crtl1 and aggrecan gene (Carulli et al., 2010; Giamanco et al., 2010; Rowlands et al., 2018), will help to elucidate the PNNs role in the healthy and injured spinal cord. An enhanced knowledge of spinal PNNs would facilitate the development of more effective and targeted strategies to properly treat SCI and other neuronal disorders, where both protection of synaptic integrity and controlled plasticity are needed.

## Author Contributions

This review was written by JS-V, with editing from ML and EU. All authors contributed to the article and approved the submitted version.

## Funding

This study was funded by the Fundació La Marató-TV3 (TV3-201736-30-31).

JS-V and EU's research was supported by funds from CIBERNED and TERCEL Networks, co-funded by European Union (ERDF/ESF, investing in your future). JS-V holds a predoctoral fellowship of the AGAUR, Secretaria d'Universitats i Recerca del Departament d'Empresa i Coneixement de la Generalitat de Catalunya, cofunded by European Social Funds. ML is supported by funds awarded from the National Institutes of Health (R01 NS104291), Wings for Life and the Lisa Dean Moseley Foundation.

## Conflict of Interest

The authors declare that the research was conducted in the absence of any commercial or financial relationships that could be construed as a potential conflict of interest.

## Publisher's Note

All claims expressed in this article are solely those of the authors and do not necessarily represent those of their affiliated organizations, or those of the publisher, the editors and the reviewers. Any product that may be evaluated in this article, or claim that may be made by its manufacturer, is not guaranteed or endorsed by the publisher.
